# Impact of load expectations on neuromuscular and postural strategies during a freestyle lifting task in individuals with and without chronic low back pain

**DOI:** 10.1371/journal.pone.0246791

**Published:** 2021-02-08

**Authors:** Catherine Daneau, Charles Tétreau, Thomas Deroche, Camille Mainville, Vincent Cantin, Martin Descarreaux

**Affiliations:** 1 Department of Anatomy, Université du Québec à Trois-Rivières, Québec, Canada; 2 Department of Human Kinetics, Université du Québec à Trois-Rivières Québec, Canada; 3 Complexité, Innovation, Activités Motrices et Sportives (CIAMS), Université Paris-Sud, Université Paris-Saclay, Orsay Cedex, France; 4 Complexité, Innovation, Activités Motrices et Sportives (CIAMS), Université d’Orléans, Orléans, France; Texas A&M University, UNITED STATES

## Abstract

**Objective:**

This study aimed to investigate how load expectations modulate neuromuscular and postural strategies in the anticipation of a freestyle lifting task with varying expected loads in individuals with and without chronic low back pain (cLBP).

**Methods:**

Forty-seven participants, 28 with cLBP pain and 19 without, were recruited and completed a series of freestyle lifting trials (3 sets of box lifted for a total of 36 lifts). Verbal cues were used to modulate their expectations about the boxes’ weight: no expectation, lighter or heavier load expectations. Following each set, participants rated their perceived exertion on a visual analog scale. During the lifting protocol, kinematics (time to maximal flexion, angular velocity and joint angles), electromyography muscle activity (erector spinae and quadriceps) and center of pressure displacement were simultaneously recorded.

**Results:**

Results showed that time to maximal knee flexion was modulated by load expectations in both groups (mean lighter load expectations = 1.15 ± 0.32 sec.; mean heavier load expectations = 1.06 ± 0.31 sec.). Results also showed a load expectations X group interaction for that time to maximal hip and lumbar flexion. Time to maximal hip flexion decreased with heavier load expectations (mean lighter load expectations = 1.20 ± 0.36; mean heavier load expectations = 1.16 ± 0.33) for cLBP only. Time to maximal lumbar flexion increased with heavier load expectation (mean lighter load expectations = 1.41 ± 0.27 sec.; mean lighter load expectations = 1.46 ± 0.29 sec.) for participants without LBP. However, no difference in lumbar, hip nor knee angles were observed between groups or conditions. Results highlighted significant load expectation effects for erector spinae electromyography activity, as lower muscle activations was observed for both groups with heavier load expectations (mean = 0.32 ± 0.15), compared to lighter load expectations (mean = 0.52 ± 0.27). Force plates analyses did not reveal any significant load expectation effects.

**Conclusion:**

Present findings showed that load expectations modulate movement strategies and muscle activation similarly but not identically in individuals with chronic low back pain and healthy adults during freestyle lifting. Results of the present study partially differ from previous studies and suggest only minor differences in lifting strategies between healthy individuals and individuals with cLBP experiencing low level of pain and disability. More studies are needed to investigate the potential role of load expectations in the development and persistence of chronic low back pain.

## Introduction

According to the 2015 Global Burden of Disease Study, low back pain (LBP) is ranked first as the most disabling musculoskeletal problem in multiple countries [1]. LBP is classified into three different types of pain durations, the chronic form (cLBP) of which is described by pain present for more than twelve weeks [2]. LBP is characterized by ache or muscle tension in the lumbopelvic region, below the costal margin and above the inferior gluteal folds [2], with consequential functional impairment [3] frequently associated to reduced function of both the lumbar spine and hips [4]. Lifting task has been widely used to observe the kinematic characteristics of the lumbopelvic region and knees [5], and the manner in which these regions are coordinated in chronic LBP, in comparison with healthy individuals [6–8].

The National Institute for Occupational Safety and Health’s Work Practice Guide for Manual Lifting reported that vertical positioning of the load at the beginning of the lifting task is one of the determinants to take into account for weight limit [9]. In fact, the location of the load has an impact on the range of motion and lifting capacity during a lifting task [10]. Many lifting behaviors seem to differ between individuals with cLBP and those without. Previous studies showed, for healthy individuals, a significant association between hip movement during trunk extension from full unloaded flexion and lumbar movement during various lifting positions [11] and a significant influence of weight and trunk flexion angle on lumbar/pelvic angle ratios [12]. In contrast, individuals with cLBP had a decrease in lumbar flexion and an increase in thoracic flexion [6, 7], a greater knee flexion and smaller hip flexion [5] and a significant lower peak velocity of hip and lumbar region [13].

Physiological factors and processes, studied as correlates of LBP in lifting task [14, 15], could drive the kinematic and behavioral characteristics of individuals experiencing LBP. The activation of trunk muscles also has its importance while realizing a lifting task. Since muscles are responsible for force production [16], in case of a lifting task, they have to be engaged even before the first phase of the movement. Individuals with LBP seem to experience greater spine compression and shear forces than individuals without LBP when performing a lifting task [15]. Moreover, our pilot study obtained similar results, with significant differences between individuals with and without LBP for erector spinae at L3-L4 and quadriceps (vastus lateralis) muscle activity when performing a freestyle lifting [14].

While some factors may play a role during the entire movement, some factors prepare an individual to realize a specific movement before it is initiated. According to biomechanics, a postural strategy is set in place prior any movement, as described by the anticipatory postural adjustments (APA) [17, 18]. It serves three main goals: (1) minimizing the disturbance of balance during movement and (2) of postural orientation of body segments and (3) assisting the movement performance in terms of velocity or force [19]. Growing scientific evidence suggest that APA strategies are altered in LBP patients. For instance, patient with LBP exhibit a reduced capacity to adapt trunk muscle recruitment to an upcoming task [20]. A review by Ruhe et al. (2011) also reports that individuals with LBP presented greater center of pressure (CoP) displacement than healthy individuals, resulting in greater postural standing instability [21]. Since these altered adaptative strategies may play a role in the development and maintenance of chronic low back pain, targeted interventions have been developed. Among these, sensory motor training seem promising and although they induce little changes on APA strategies, results indicate a positive impact on pain and function [20, 22].

LBP is also associated with psychological factors, including anxiety and pain behaviors, resulting in an outward display of pain and guarding [3]. A previous study showed that individuals with cLBP who have a higher score on Pain Catastrophizing Scale (PCS) felt more disabled because of their pain and suffered more from psychological distress [23]. Moreover, Pfingsten et al. (2001) showed that when pain anticipation was induced by instruction, levels of behavioral performance was lower, and pain intensity and related fear were increased during a single leg-flexion task [24]. This can suggest that fear of pain, as a protective strategy, might influence the normal execution of a freestyle lifting task for LBP individuals compared to healthy ones. Our pilot study has observed a relationship between load expectations and perceived exertion responses in freestyle lifting [14]. As reported in this pilot study, modulation of load expectations triggered modification in the individuals’ perception of exertion which result in an increased electromyographic activity [14]. It seems interesting to further investigate how psychological factors, including load expectations, can amplify or modulate the maladaptive behaviors observed in LBP patient.

To the best of our knowledge, the previously mentioned pilot study [14] led by our research team, was the first to investigate kinematics (range of motion and angular velocity), postural CoP displacement), physiological (muscle activity) and perceived exertion during a freestyle lifting task in a protocol inducing lower and higher load expectations in individuals with cLBP. Since preliminary results were promising, it became relevant to deepen our investigation in a larger sample by modifying kinematic assessment and focusing on the neuromechanical adaptations preceding the lift. Therefore, the goal of the present study was to investigate how load expectations modulate kinematics and neuromuscular control, during a freestyle lifting task with varying expected loads in individuals with and without cLBP.

## Materials and methods

### Participants

Participants were recruited through advertisement on the university campus, social media and an on-campus outpatient chiropractic clinic. A total of 47 participants were tested in order to compare results of patients with (28 participants) and without (19 participants) cLBP, and attention was given to matching participants’ characteristics between groups. To be included in the cLBP group, participants had to have experienced recurrent or persistent LBP for at least six months defined as pain or discomfort located below the costal margin and above the inferior gluteal folds [25], though not at a level limiting their capacity to execute the lifting protocol, the latter consisting of a total of 36 freestyle lifts of a box positioned on the ground weighting between 6.8 kg and 13.6 kg. Individuals in the non-cLBP group should not have experienced LBP in the last five years. Subjects were systematically excluded from the experiment if they were currently experimenting a flare-up episode of LBP or presented health problems such as uncontrolled hypertension, neuromuscular disease, cancer, consumption of psychotropic drugs, as well as specific LBP, including spinal stenosis, herniated disc, back operation or traumatic injury. Additionally, pregnant or breastfeeding women were also excluded. The study was carried out in accordance with the Human Research Ethics Committee of the Université du Québec à Trois-Rivières who granted ethical certification for the study (CER-15-219-07.12). All subjects provided written informed consent in accordance with the Declaration of Helsinki.

### Procedures

**Fig 1:**
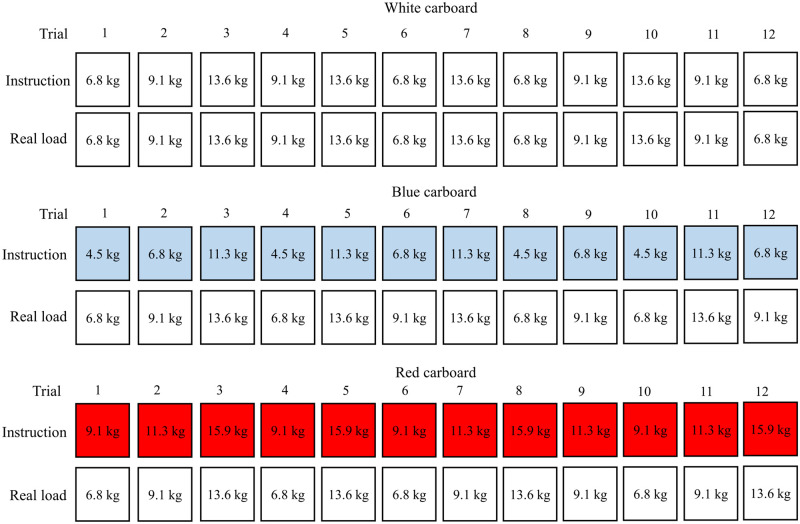
Illustration of the three sets of twelve box lifts.

This protocol builds on a pilot study conducted previously [14] to which slight adjustments were made in order to improve kinematic data collection and the modulation of expectations related to the weight of the boxes to be lifted. Patients were invited to the University’s Neuromechanics and Motor Control Laboratory; their commitment included a single visit. After the initial measures, including anamnesis, (self-reported) weight and height, questionnaires filling (information on catastrophizing, kinesiophobia, anxiety and functional limitations) and rating of pain on a visual analog scale, participants were instrumented with surface electromyography and kinematics sensors. Maximal voluntary contraction (MVC) of the lumbar erector spinae and of the quadriceps were recorded for normalization before the lifting protocol started. The latter consisted of three sets of twelve box lifts (Fig 1) from the ground, for which the participants received the following verbal instructions: “Lift the box as you would in everyday life while holding both handles located on its sides as naturally as possible”. Each lift started in a standing position. At a self-chosen pace, the participants then leaned forward to reach the box, grabbed it, executed a full knee and hip extension to regain a standing position for two seconds, bringing the box with them, and then repositioning the box on the ground. For each lift, the prelifting phase corresponded to the time period before the box was lifted from the force plate. A 30 seconds rest period was allowed between each trial. A total of 9 identical hard plastic boxes measuring 42 x 33 x 24 cm (length x width x height) with handles on each side, in which different combination of weight plates, not visible to the participant, were firmly fixed to obtain the desired mass were used for this protocol. The first set of twelve repetitions consisted of four lifts of a 6.8-, 9.1- and 13.6-kg boxes on which a white cardboard was affixed to indicate the real weight that was lifted. This first set can be considered as familiarization since only the data from the second and third sets, in which load expectations were manipulated, were analyzed. For the second and third sets of repetitions, randomly counterbalanced between participants, the weights of the lifted boxes were identical however, verbal cues were used in combination to visual information (blue and red cardboards) to reinforce the modulation of expectations about the weight of the boxes, since these two strategies have been demonstrated to have a cumulative effect [26]. In the heavier load expectations set, a red cardboard placed on the boxes’ lids indicated a weight of 2.3 kg higher than the real boxes’ weight (for example, the red cardboard on the 9.1 kg box indicated 11.4 kg). On the opposite, a weight of 2.3 kg lower than the real boxes’ weight was written on a blue cardboard for the lighter load expectations set (for example, the blue cardboard on the 9.1 kg box indicated 6.8kg). The weights of the boxes were chosen according to the literature evaluating similar task with a LBP population [7], and because our pilot study showed that weights of 2.3, 5.7 and 9.1 kg cause little exertion to both people with and without LBP [14]. Each set of twelve repetitions lasted around 7 to 8 minutes and participants were given a 10-minute break between each set in order to help limit pain increase for participants with LBP and to separate the different sets by enough time so that participants could hardly realize that they were in fact lifting the exact same weights in the three sets.

### Measurements

#### Kinematics

Kinematics data were collected to evaluate anticipation and lifting strategies. A motion analysis system (Optotrak Certus; Northern Digital, Waterloo, Ontario, Canada) was used in combination with four kinematics markers placed on anatomical landmarks, three virtual kinematics marker and two clusters of active markers all placed on the subject’s right side. The exact location of these markers were 1) kinematics markers: lateral malleolus, femur’s lateral condyle, T1 and T11, 2) virtual kinematics markers: anterosuperior iliac spine (ASIS), posterosuperior iliac spine (PSIS) and great trochanter and 3) clusters of active markers: centered on mid-sacrum and lateral mid-thigh. These markers allowed the creation of three joint angles: the knee angle (lateral malleolus, femur’s lateral condyle and great trochanter), the hip angle (the intersection between the thigh segment created by the femur’s lateral condyle and the great trochanter, and the pelvis segment created by ASIS and PSIS) and the lumbar spine angle (intersection between lumbar segment created by L1 and T11 and the pelvis segment created by ASIS and PSIS). This setup was chosen to help tracking markers that could disappear because of the arm movement throughout the lifting task as experimented in the pilot study [14]. Time to maximal flexion, angular velocity and joint angles were calculated using Matlab (MathWorks, Natick, Massachusetts). All kinematics recordings were made at 100Hz.

#### Electromyography

Surface electromyography (sEMG), consisting of bipolar disposable surface Ag/AgCl electrodes (Bortec Biomedical, Calgary, Alberta, Canada), was used to register muscle activation strategy for lumbar erector spinae at L3 and at vastus lateralis. Skin was prepared to reduce skin impedance by shaving body hair, gently abrading with fine-grade sandpaper (Red Dot Trace Prep; 3M, St. Paul, Minnesota) and wiping with alcohol swabs. Electrodes were then placed bilaterally, and a ground was positioned on the left ASIS completed the setup. A Delsys sEMG sensor recorded data with a common mode rejection ratio of 92dB at 60Hz and an input impedance of 10^15^ Ω (model DE2.1; Delsys, Inc., Boston, Massachusetts). Data were sampled at 1000Hz with a 12-bit A/D converter (PCI 6024E; National Instruments, Austin, Texas). After the data collection with LabView (National Instruments, Austin, Texas), the sEMG signals were processed by Matlab (MathWorks, Natick, Massachusetts).

#### Maximal voluntary contraction

Participants realized two or three MVC of the back extensor muscles on a Roman chair inclined at 45 degrees [27]. To ensure a proper MVC, the participant had to perform a third MVC if his second trial was higher than his first trial and encouragement were provided for each trial in both groups. In a prone position, their iliac crests were aligned with the side of the cushion and their trunk maintained unsupported [27]. They performed the MVC against a belt installed over their shoulders (adjusted for each participant to ensure a straight position), attached to a load cell (NTEP-87-057A3 class III; Artech, Riverside, CA) and permanently fixed to the ground by a cable. The MVC was composed of two phases: (1) the participants maintained a horizontal position during five seconds then (2) they lifted their trunk until they felt a tension in the belt and performed the maximal isometric back extensions for five seconds. Participants also realized three MVC of the quadriceps seated on a bench, knees at 90 degrees and their feet unsupported. They performed the MVC against a strap placed on the tibia by doing an extension of both knees at the same time for five seconds without using their arms for help. Participants were strongly verbally encouraged by team members while performing MVC and rest periods of 60 seconds were allowed between each MVC trial. After a five minutes seated break, the lifting protocol started.

#### Force plates

Two force plates (AMTI: Model OR6-5-2000, Advanced Mechanical Technology, Inc., Newton, MA, USA and Bertec: model 4550–08, Bertec Corporation, Columbus, Ohio, USA) were positioned side by side. The first one was used for boxes, and detected the onset of lifting movements, while the second one was used to calculate the participant’s center of pressure position. Floor markers were used to ensure that both the boxes and the participant’s foot were always positioned similarly for each trial. Only vertical forces were collected from the box force plate (to detect the onset of the lift), as opposed to the foot force plate from which vertical, lateral and anteroposterior forces and moments were recorded to assess the CoP displacement and mean CoP velocity. All force plate recordings were made at 100Hz.

#### Self-administered questionnaire

Before the beginning of the experiment, all participants filled the French validated translation of the fear-avoidance belief questionnaire (FABQ) [28], the pain catastrophizing scale (PCS) [29], the Tampa scale of kinesiophobia (TSK) [30] and the state-trait anxiety inventory form Y (STAI-Y) [31]. Participants with cLBP also completed the Oswestry disability index (ODI) [32], reported the number of years living with LBP and rated their actual pain on a visual analog scale (VAS) ranging from 0 to 10, representing respectively no pain and the most intense imaginable pain [33].

#### Perceived exertion

In order to determine if the load expectation manipulation protocol yielded a significant impact on participant perception, global perceived exertion was collected at the end of each of the three set. A category-ratio scale anchored at 0 representing no effort at all and 10 representing a maximal effort was used. A previous pilot study [14] indicated that this tool was effective at detecting statistical differences, although clinically small since global exertion ratings were generally very low.

### Data analysis

Each of the descriptive variable (Table 1) were compared using unpaired Student’s T-test to assess between group differences. sEMG data were filtered digitally by a 10- to 450 Hz band-pass, zero-lag, and fourth-order Butterworth filter. Modulation of the sEMG amplitude was calculated by using the root mean square (RMS) value across 500-ms windows before lifting for all investigated muscles. Normalized RMS sEMG values (nRMS) were obtained by dividing this 500-ms mean RMS by the 500-ms mean RMS calculated during the peak of the highest MVC of the respective muscle. For all subsequent analyses, nRMS values were used.

**Table 1:** Participants’ characteristics.

	With cLBP		Without cLBP		
	N = 28		N = 19		
Characteristics	Mean	SD	Mean	SD	*P*
Age (years)	36.46	16.01	29.16	8.93	.08
Weight (kg)	71.36	15.24	66.58	12.53	.26
Height (m)	1.70	0.08	1.70	0.10	.89
Pain pre-test (VAS /10 cm)	1.88	2.19	-	-	.00 **
Pain post-test (VAS /10 cm)	2.05	2.50	-	-	.00 **
Duration of LBP (years)	10.05	9.11	0.00	0.00	.00 **
ODI (/100)^a^	10.93	6.90	0.63	1.50	.00 **
PCS (/52)^b^	9.71	6.85	7.1	7.96	.24
TSK (/68)^c^	33.14	6.55	29.84	5.71	.08
FABQw (/42)^d^	7.54	9.85	3.47	7.11	.13
FABQpa (/24)^e^	7.05	5.03	3.98	4.91	.04 *
STAI-Y A5 (/80)^f^	27.21	7.10	28	7.11	.71
STAI-Y (/80)^f^	33.30	8.41	33.47	7.11	.94

cLBP, chronic low back pain; SD, Standard deviation; VAS, Visual analog scale.

^a^ Oswestry Disability Index (ODI) [32].

^b^ Pain Catastrophizing Scale (PCS) [29].

^c^ Tampa Scale of Kinesiophobia (TSK) [30].

^d^ Fear-avoidance Beliefs Questionnaire work subscale (FABQw) [28].

^e^ Fear-avoidance Beliefs Questionnaire physical activity subscale (FABQpa) [28].

^f^ State-Trait Anxiety Inventory form Y (STAI-Y) [31].

*p < .05.

**p < .001.

Time to maximal flexion was obtained by calculating the time from the onset to the end of the flexion phase individually for each of the knee, hip and lumbar joint. Joint angles for the knee, hip and lumbar joint were calculated by deducting the angle in the most flexed position minus the angle in the initial upright position. Angular velocities were computed by dividing joints angles by time to maximal flexion of the respective joint. CoP displacement was evaluated in the antero-posterior axis only and was calculated as the difference in positioning between CoP in the initial upright position and CoP before the onset of the lift. Mean CoP velocity was calculated using the first derivate of CoP displacement. The four trials per condition were average for each variable and the mean was used for all statistical analysis. The analyses were performed using STATISTICA statistical package version 10 (Statsoft, Tulsa, OK).

The Shapiro-Wilk and the Kolmogorov-Smirnov tests were used to assess each variable for normality. Mixed model ANOVAs (2x2) were conducted using expectations (lighter and heavier loads) and groups (participants with cLBP and those without cLBP) for each of the following variables: Perceived exertion (Borg score), lumbar erector spinae and quadriceps muscle activity (nRMS), kinematics (time to maximal flexion, angular velocity and angles for the knee, hip and lumbar joint) and force plate data (CoP displacement and mean CoP velocity). Post hoc analyses (LSD test) were conducted whenever necessary. For all analyses, the level of significance was set at *p* ≤ 0.05.

## Results

From the 47 original participants, 7 were excluded of the kinematics analysis because of high loss of data. One participant did not complete all the freestyle liftings due to high apprehension regarding the lifting of heavier weights.

### Participants’ characteristics

Both groups were similar in terms of age, weight, height, perceived exertion and different questionnaires (PCS, TSK, FABQw and both IASTA). As expected, pain pre- and post-test, as well as duration of LBP (years) were significantly different between both groups. Means pain ratings and ODQ scores indicated that individuals with LBP presented mild LBP and minimal disability levels [34]. Participants’ characteristics (with and without cLBP) are presented in Table 1 and perceived exertion is presented in Table 2.

**Table 2:** Participants’ perceived exertion.

	Df	MSE	F	P	η^2^_p_
Group	1, 44	1.81	0.24	0.62	0.005
Load expectations	1, 44	6.76	14.84	<0.001**	0.25
Load expectations x Group	1, 44	0.35	0.00	0.38	0.02

df, degrees of freedom; MSE, mean square error; F, F statistic;

**p < .001,

η^2^_p_, partial eta squared.

### Kinematics

#### Time to maximal flexion and joint angle

Results showed a significant load expectations X group interaction (F(1,42) = 4.23, *p* = 0.046, η^2^_p_ = 0.09) for time to maximal hip flexion. Post-hoc analyses revealed that, for cLBP participants, the time to maximal hip flexion decreased with heavier load expectations compared to lighter load expectations (Mean _lighter load expectations, with LBP_ = 1.20 ± 0.36 sec.; _Mean heavier load expectations, with LBP_ = 1.16 ± 0.33 sec.) compared to participant without LBP for which such decreased was not observed (Mean _lighter load expectations, without LBP_: 1.27 ± 0.31 sec.; Mean _heavier load expectations, without LBP_ = 1.28 ± 0.27 sec.). Results showed a significant load expectations X group interaction (F(1,40) = 5.83, *p* < 0.020, η^2^_p_ = 0.13) for time to maximal lumbar flexion. Post-hoc analyses revealed that participants without pain increased their time for maximal lumbar flexion with heavier load expectations compared to lighter load expectations (Mean _lighter load expectations, without LBP_ = 1.41 ± 0.27 sec.; Mean _lighter load expectations, without LBP_ = 1.46 ± 0.29 sec.). In contrast, participant with LBP showed no difference between the lighter and heavier load expectation conditions (Mean _lighter load expectations, with LBP_ = 1.39 ± 0.31 sec.; Mean _heavier load expectations, with LBP_ = 1.36 ± 0.27 sec.). Results showed a significant main effect of load expectations (F(1,41) = 71.14, *p* < 0.001, η^2^_p_ = 0.63) for time to maximal knee flexion, indicating that time to maximal knee flexion was higher with lighter load expectations than heavier load expectations (Mean _lighter load expectations_ = 1.15 ± 0.32 sec.; Mean _heavier load expectations_ = 1.06 ± 0.31 sec.). No significant differences were found concerning the hip, lumbar or knee angles. Table 3 presents the main effects and interactions for hip, lumbar and knee time to maximal flexion and angles.

**Table 3:** Main effects and interactions for hip, lumbar and knee time to maximal flexion and angles.

	Df	MSE	F	P	η^2^_p_
Time to maximal hip flexion					
Group	1, 42	0.18	0.98	0.33	0.02
Load expectations	1, 42	0.003	1.07	0.31	0.02
Load expectations x Group	1, 42	0.02	4.23	0.046*	0.09
Time to maximal lumbar flexion					
Group	1, 40	0.06	0.52	0.47	0.01
Load expectations	1, 40	0.002	0.30	0.59	0.01
Load expectations x Group	1, 40	0.03	5.83	0.02*	0.13
Time to maximal knee flexion					
Group	1, 41	0.10	0.78	0.38	0.02
Load expectations	1, 41	0.17	71.14	<0.001**	0.63
Load expectations x Group	1, 41	0.009	3.79	0.058	0.08
Hip angle					
Group	1, 42	2.0	0.0009	0.93	0.0002
Load expectations	1, 42	0.0	0.009	0.92	0.0002
Load expectations x Group	1, 42	0.1	0.02	0.89	0.0005
Lumbar angle					
Group	1, 40	227.5	0.60	0.44	0.01
Load expectations	1, 40	8.3	1.51	0.23	0.04
Load expectations x Group	1, 40	8.1	1.48	0.23	0.04
Knee angle					
Group	1, 41	2490.0	3.79	0.0585	0.08
Load expectations	1, 41	12.9	2.41	0.13	0.06
Load expectations x Group	1, 41	0.1	0.02	0.89	0.0004

df, degrees of freedom; MSE, mean square error; F, F statistic;

*p < .05;

**p < .001;

η^2^_p_, partial eta-squared.

#### Angular velocity

**Fig 2:**
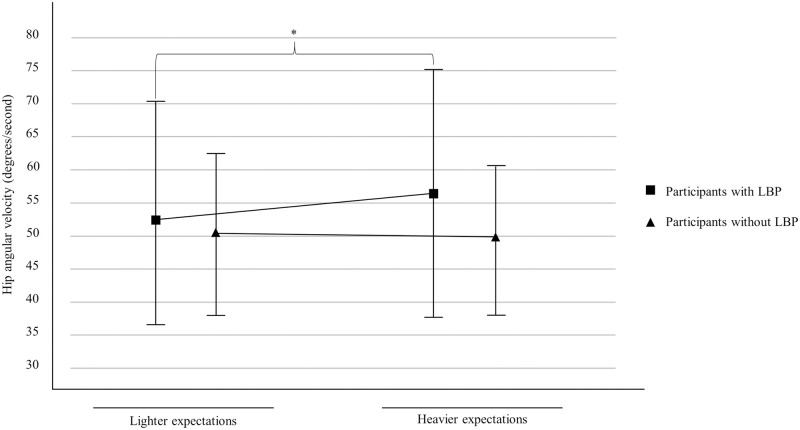
Load expectations X group interaction for hip angular velocity. cLBP, chronic low back pain; * *p* < .05. Bars indicate standard deviation.

Results showed a significant load expectations X group interaction (F(1,42) = 7.62, *p* = 0.009, η^2^_p_ = 0.15) for hip angular velocity (Fig 2). Post-hoc analyses revealed that hip angular velocity, for cLBP participants only, was significantly higher for heavier load expectations compared to lighter load expectations (Mean _lighter load expectations, with LBP_ = 53.71 ± 7.19; Mean _heavier load expectations, with LBP_ = 56.92 ± 18.65; Mean _lighter load expectations, without LBP_ = 50.93 ± 12.80; Mean _heavier load expectations, without LBP_ = 49.13 ± 11.00). Table 4 presents the main effects and interactions for hip, lumbar and knee angular velocity.

**Table 4:** Main effects and interactions for hip, lumbar and knee angular velocity.

	Df	MSE	F	P	η^2^_p_
Hip					
Group	1, 42	593.5	1.33	0.25	0.03
Load expectations	1, 42	10.5	0.60	0.44	0.01
Load expectations x Group	1, 42	133.7	7.62	0.01*	0.15
Lumbar					
Group	1, 38	6.67	0.03	0.87	0.0007
Load expectations	1, 38	4.45	0.70	0.41	0.18
Load expectations x Group	1, 38	0.44	0.07	0.79	0.002
Knee					
Group	1, 39	1761.3	1.85	0.18	0.05
Load expectations	1, 39	2.2	0.14	0.71	0.004
Load expectations x Group	1, 39	23.2	1.48	0.23	0.04

df, degrees of freedom; MSE, mean square error; F, F statistic;

*p < .05;

η^2^_p_, partial eta-squared.

### Electromyography

Analysis of sEMG data showed a significant main effect of load expectations (F(1,43) = 63.49, *p* < 0.001, η^2^_p_ = 0.60) during the prelifting phase. It revealed that lumbar erector spinae sEMG activity was higher with lighter load expectations compared to heavier load expectations (Mean _lighter load expectations_ = 0.52 ± 0.27; Mean _heavier load expectations_ = 0.32 ± 0.15). However, although mean quadriceps sEMG activity seem to increase with heavier load expectations compared to lighter load expectation (Mean _lighter load expectations_ = 0.87 ± 0.39; Mean _heavier load expectations_ = 0.89 ± 0.40), results did not reach statistical significance (F(1,44) = 3.55, *p* = 0.066, η^2^_p_ = 0.07). Table 5 presents the main effects and interactions for the lumbar erector spinae and the quadriceps sEMG activity.

**Table 5:** Main effects and interactions for the lumbar erector spinae and the quadriceps sEMG activity.

	Df	MSE	F	P	η^2^_p_
Lumbar erector spinae–prelifting					
Group	1, 43	0.01	0.25	0.62	0.006
Load expectations	1, 43	0.87	63.49	< 0.001**	0.60
Load expectations x Group	1, 43	0.01	0.73	0.40	0.02
Quadriceps–prelifting					
Group	1, 44	0.0001	0.0004	0.99	0.000008
Load expectations	1, 44	0.006	3.55	0.07	0.07
Load expectations x Group	1, 44	0.002	0.40	0.53	0.009

df, degrees of freedom; MSE, mean square error; F, F statistic;

*p < .05;

**p < .001;

η^2^_p_, partial eta-squared.

### Center of pressure displacement and velocity

No main effects or interactions were found for the prelifting CoP displacement or for mean CoP velocity. Table 6 presents the main effects and interactions for CoP posterior displacement and mean CoP velocity.

**Table 6:** Main effects and interactions for CoP posterior displacement and mean CoP velocity.

	Df	MSE	F	P	η^2^_p_
Posterior displacement					
Group	1, 44	171.80	2.13	0.15	0.05
Load expectations	1, 44	0.65	0.07	0.80	0.001
Load expectations x Group	1, 44	8.06	0.80	0.37	0.02
Mean velocity					
Group	1, 44	17.97	0.56	0.46	0.01
Load expectations	1, 44	0.49	0.22	0.64	0.005
Load expectations x Group	1, 44	0.11	0.05	0.82	0.001

df, degrees of freedom; MSE, mean square error; F, F statistic;

*p < .05;

η^2^_p_, partial eta-squared.

## Discussion

The present study aimed at improving our understanding of mechanisms involved in neuromuscular and postural control during free lifting tasks by comparing motor and postural strategies preceding a freestyle lifting task in healthy individuals and individuals with cLBP. To our knowledge, this is the first study that quantifies the effects of load expectations on prelifting postural adjustments in a freestyle lifting task in healthy adult participants and participants with cLBP.

As expected, the protocol successfully modified load expectations in both groups, which is consistent with previous studies that have used similar visual and verbal cues to modify expectations about lifting weights [14] or expectation of a painful stimulation [35, 36]. In the present study, perceived exertion was significantly different between the two sets of instructions for the same weights lifted indicating that load expectations modulated the perception of efforts during lifting.

Results of the study showed that prelifting strategies were similar in individuals with and without cLBP. In fact, there was no significant differences in kinematics, electromyography and center of pressure displacement between individuals with and without cLBP pain as they prepared to lift boxes of varying weights. Such results appear to be in contradiction with the broad-based consensus that cLBP patients present altered anticipatory and compensatory postural adjustments in various tasks [37, 38]. However, a recent meta-analysis showed strong evidence of a delayed muscle responses in individuals with low back pain but far less evidence suggesting alterations in CoP and kinematics. The authors concluded that changes in timing may not lead directly to impaired movement control [38].

Over the years, several studies have brought up results suggesting adaptations in anticipatory postural adjustments of cLBP patients. For instance lower and higher muscle activity of the deep and superficial trunk muscles in supine leg raise [20], delayed and impaired deep trunk muscle activation preceding rapid arm flexion [39] and changes in kinematic strategies in preparation to a lifting task [5] have all been reported. This latter study is particularly relevant, since direct comparison can be made with the present study results, as some kinematic variables were assessed in a similar context. Their results indicate that when lifting a load, participants with LBP, compared to control participants, increase their knee flexion by about 10° while reducing hip flexion by about 8°. Angular displacement were similar for both groups and throughout all conditions in our study and kinematic group differences were rather observed in angular velocity. Such differences between the two studies may be due to instructions given to participants. Rudy et al (2003) instructed their participants to perform multiple lifting for 20 minutes with limited rest period [5]. The number of lifting was significantly lower in the LBP patient group indicating that participants may have prioritize specific strategies (e.g. minimizing effort and energetic cost) over others. Pain, fatigue and possible de-conditioning probably had limited impact on our results, compared to the results published by Rudy et al. A recent systematic review exploring the differences in kinematics and muscle activity of the trunk and lower limbs in people with and without LBP during freestyle lifting tasks showed that individuals with LBP move differently than healthy individuals [40]. However, the review showed several inconsistencies across studies and trends toward greater changes in strategies in individuals who present more severe LBP. The results of the current project are therefore at least partially in line with findings of this review. Indeed, it is hypothesized that the modest differences observed between our LBP and control groups may be explained by the fact that mild LBP patients presenting low level of disability and disturbance of psychological factors related to movement may to behave more similarly to people without LBP than to people with severe LPB.

The present study showed that cLBP participants, in submaximal non-fatiguing lifting tasks are able to perform similarly to healthy adult participants. Additionally, modulating load expectations yielded changes in lifting strategies of mostly medium and large effect sizes suggesting that these changes are more than trivial. These changes, however, were consistent in both participants with and without low back pain. For both groups, erector spinae sEMG activity showed less activity while quadriceps sEMG activity leaned toward increased activity when expectations of heavier loads were triggered. Combined with knee kinematics changes (time to maximal flexion), such results suggest that expectations regarding a weight to lift trigger similar adaptations in patients experiencing mild cLBP and healthy individuals. Although no difference was noted in joint angles, probably due to high heterogeneity and relatively low loads, the current observations seems congruent with the broadly accepted idea that deeper knee flexion during squatting technique contributes to the unloading of hip and the back articulations [41]. Interestingly, minor differences were found in a few kinematic variables indicating that participants with LBP respond similarly, but not exactly as healthy individuals to load expectations. Although small between group differences in hip angular velocity, time to maximal flexion of hip and lumbar joints were observed when participants expected heavier loads, the clinical impact of such differences remains unknown but is probably trivial. As mild differences were observed in psychological factors between LBP and non-LPB groups, mainly fear-avoidance behaviors, it could be hypothesized that they can be a contributing factor to explain why load expectations yielded these minor kinematics adaptations in cLBP patients.

### Limitations

Some limitations need to be considered when interpreting the results of the current study. First, a few main and interactions did not reach statistical significance by a very thin margin suggesting that a larger sample may have yielded group differences in knee flexion related variables. In addition, our sample of participants with cLBP was recruited and included in the study only if they were not in an active episode of LBP. This led to the recruitment of functional participants with cLBP for whom disability and pain scores were relatively low at the time of testing. Results should therefore be generalized with caution to other cLBP populations.

Secondly, although weights were increased by 50% following our pilot study, the self-perceived exertion remained relatively low during our testing protocol. More challenging tasks may lead to higher effects and expose differences between healthy and cLBP individuals that were not present in this study.

## Conclusion

Although the present findings only partially concur with previous evidence, they suggest that healthy individuals and cLBP patients with low level of disability and pain use similar movement strategies while performing a freestyle lift of a box from the ground to hip height. The present study showed that load expectations regarding lifting conditions can be successfully modulated in experimental laboratory settings. Inducing load expectations modulated kinematics and muscle activation similarly in participant of both groups. Although, load expectations triggered minor differences in the lifting strategies between groups our main findings suggest that LBP patients with low levels of disability and pain use similar lifting strategies even when dealing with misleading load expectations. As such results suggest that load expectations alter lifting strategies both in low back pain patients and pain free controls, more studies are needed to investigate whether these movement alterations play a role in the development and persistence of chronic low back pain.
